# Mannose enhances intestinal immune barrier function and dextran sulfate sodium salt-induced colitis in mice by regulating intestinal microbiota

**DOI:** 10.3389/fimmu.2024.1365457

**Published:** 2024-03-11

**Authors:** Yi Yang, Qiming Ma, Qingyu Wang, Lifeng Zhao, Hengshan Liu, Yanjun Chen

**Affiliations:** ^1^ Department of Bariatric Surgery, The First Affiliated Hospital of Jinan University, Guangzhou, China; ^2^ Department of Pharmacy, Affiliated Cancer Hospital of Inner Mongolia Medical University, Peking University Cancer Hospital Inner Mongolia Hospital, Hohhot, China; ^3^ Department of Emergency and trauma, Yichang Central People’s Hospital, Yichang, Hubei, China; ^4^ Department of Anesthesiology, The First Affiliated Hospital of Jinan University, Guangzhou, China

**Keywords:** colitis, intestinal microbiota, mannose, intestinal immune, inflammation

## Abstract

**Background:**

Inflammatory bowel disease (IBD) greatly affects human quality of life. Mannose has been reported to be used to treat IBD, but the mechanism is currently unknown.

**Methods:**

C57/BL mice were used as research subjects, and the mouse acute colitis model was induced using dextran sulfate sodium salt (DSS). After oral administration of mannose, the body weights and disease activity index (DAI) scores of the mice were observed. The colon lengths, histopathological sections, fecal content microbial sequencing, colon epithelial inflammatory genes, and tight junction protein Occludin-1 expression levels were measured. We further used the feces of mice that had been orally administered mannose to perform fecal bacterial transplantation on the mice with DSS-induced colitis and detected the colitis-related indicators.

**Results:**

Oral administration of mannose increased body weights and colon lengths and reduced DAI scores in mice with DSS-induced colitis. In addition, it reduced the expression of colon inflammatory genes and the levels of serum inflammatory factors (TNF-α, IL-6, and IL-1β), further enhancing the expression level of the colonic Occludin-1 protein and alleviating the toxic response of DSS to the intestinal epithelium of the mice. In addition, gut microbial sequencing revealed that mannose increased the abundance and diversity of intestinal flora. Additionally, after using the feces of the mannose-treated mice to perform fecal bacterial transplantation on the mice with DSS-induced colitis, they showed the same phenotype as the mannose-treated mice, and both of them alleviated the intestinal toxic reaction induced by the DSS. It also reduced the expression of intestinal inflammatory genes (TNF-α, IL-6, and IL-1β) and enhanced the expression level of the colonic Occludin-1 protein.

**Conclusion:**

Mannose can treat DSS-induced colitis in mice, possibly by regulating intestinal microorganisms to enhance the intestinal immune barrier function and reduce the intestinal inflammatory response.

## 1 Introduction

Inflammatory bowel diseases (IBDs), including Crohn’s disease and ulcerative colitis, are most typically characterized by the development of progressive, chronic, and relapsing inflammation in the gastrointestinal tract response. It is related to a variety of genetic and environmental factors (1, 2) and greatly affects human quality of life (3). The primary feature of IBD is disruption of intestinal homeostasis that includes dysregulated immune responses, eating disorders, genetic susceptibility, intestinal microbiota imbalance, and barrier damage (4, 5). Until now, the pathogenesis of IBD has not been fully understood. However, intestinal mucosal dysfunction and uncontrolled immune responses mediated by intestinal epithelial cells and immune cells are important factors in its occurrence (6, 7), and this has become gradually accepted as a mainstream view. In recent years, the prevalence of IBD has increased, with a troubling increasing trend among younger people. With the rapid development of high-throughput DNA sequencing and bioinformatics, it is believed that the impaired homeostasis and functional changes in intestinal flora and their metabolites play an important role in the pathogenesis of IBD (8). Furthermore, intestinal microbial disorders are well known to the public as a new pathogenic concept. Intestinal microorganisms play an important role in the intestine, including the shaping of the intestinal mucosal barrier and the regulation of intestinal immune function (9). Many drugs have been reported to be beneficial for IBD, but the mechanisms remain unclear. Some researchers have proposed the idea that drugs regulate intestinal immunity and barrier function by regulating intestinal flora.

Mannose is a natural bioactive six-carbon monosaccharide that exists in a free state in the peels of certain plants. For example, peaches, apples, and citrus peels contain certain amounts of free mannose (10). Previous studies have shown that mannose can improve metabolic syndrome, including obesity, type 2 diabetes, and non-alcoholic fatty liver disease, by reshaping the intestinal flora (10–12). It is worth noting that there is a close correlation between people with metabolic syndrome and people with intestinal barrier disorders. In recent years, researchers have gradually paid attention to the inhibitory effect of mannose on intestinal or body inflammation, and they have widely studied mannose and consider it as promising strategy (13). However, the therapeutic mechanism of mannose for colitis remains unclear.

In this study, we first demonstrate that mannose can treat dextran sulfate sodium salt (DSS)-induced colitis in mice. It was further discovered that the therapeutic mechanism may be related to inhibiting the expression of intestinal inflammatory genes and maintaining the intestinal barrier function. In addition, we found that this therapeutic effect may have been due to mannose regulating the intestinal microbiota. Fecal bacterial transplantation further confirmed that mannose enhanced the intestinal immune barrier function by regulating intestinal microorganisms and thereby alleviated DSS-induced colitis in mice.

## 2 Materials and methods

### 2.1 Animal experiments

C57/BL mice (20 ± 2 g) obtained from the Experimental Animal Center of Jinan University were used in this study. Animals were maintained under controlled temperature (21−23°C), humidity (50%), and light (12 h light/12 h dark). All animal experiments were approved by the Experimental Animal Ethics Committee of Jinan University. DSS was dissolved in drinking water at a concentration of 3.0% (MP Biomedicals, molecular weight 36,000–50,000). The mannose drug was obtained from the Sigma-Aldrich Company (3458-28-4), and the feeding standard was 500 µg/day (per kilogram of body weight). The animal feed and drinking water were sterilized prior to use.

### 2.2 Real-time fluorescent quantitative RT-PCR

Fresh tissues were ground and homogenized, and the total RNA was extracted using the TRIzol reagent (Ambion). Amplification products were labeled using SYBR (YEASEN, 10222ES60). RT-PCR was performed using the PCR system of Bio-rad. Relative mRNA levels were calculated using the comparative threshold cycle method. The primer sequences are shown in **Table 1**.

**Table 1:** Primer information table.

Gene name	Forward	Reverse
Il-6	CCGGAGAGGAGACTTCACAGA	AGAATTGCCATTGCACAACTCTT
Il-1β	GCAACTGTTCCTGAACTCAACT	ATCTTTTGGGGTCCGTCAACT
Ifn-1γ	ATGAACGCTACACACTGCATC	CCATCCTTTTGCCAGTTCCTC
Il-2	GTGCTCCTTGTCAACAGCG	GGGGAGTTTCAGGTTCCTGTA
Il-12	TGGTTTGCCATCGTTTTGCTG	ACAGGTGAGGTTCACTGTTTCT
Tgf-β	CTCCCGTGGCTTCTAGTGC	GCCTTAGTTTGGACAGGATCTG
Muc2	AGGGCTCGGAACTCCAGAAA	CCAGGGAATCGGTAGACATCG
Gpr-41	TTCTGAGCGTGGCCTATCCA	AGACTACACTGACCAGACCAG
Gpr-43	CTTGATCCTCACGGCCTACAT	CCAGGGTCAGATTAAGCAGGAG
Gpr-109a	CTGGAGGTTCGGAGGCATC	TCGCCATTTTTGGTCATCATGT
Tnf-α	GGTGCCTATGTCTCAGCCTCTT	GCCATAGAACTGATGAGAGGGAG

### 2.3 Western blot

The radioimmunoprecipitation assay buffer (RIPA) lysis buffer was used to extract the colon epithelial tissue proteins, and subsequently, a bicinchoninic acid (BCA) protein assay kit was used to determine the protein concentration (Beyotime, Shanghai, China). The polyvinylidene difluoride (PVDF) membrane was then blocked with 5% skimmed milk powder for 2 hours. The membrane was then incubated with Occludin-1 (1:1000, ab216327) at 4°C, β-Actin (1:1000, ab8226) levels were assessed as a loading control, and then a secondary antibody (1:2000) was bound to the primary antibody for 2 hours at room temperature. Signals were captured using an emitter-coupled logic substrate (Pierce Chemical Co.). The intensity of each band was quantitatively analyzed using ImageJ software.

### 2.4 Fecal transplant

In the fecal bacterial transplantation experiment, fresh feces of mice that had been orally administered mannose were used as the research material, crushed using a grinder, and then resuspended three times in buffer, after which they were centrifuged at 6000 r/min for 10 min. The supernatant was used for intragastric administration. A total of 100 µg of feces was dissolved in 200 µL of phosphate buffered saline (PBS) solution, and each mouse was given 200 µL of the fecal microbial supernatant via intragastric administration every day.

### 2.5 Serological testing

When the mice were sacrificed, the blood of the mice was collected and centrifuged at 6000 r/min for 15 minutes, and the serum was collected for relevant serological testing, i.e., the detection of TNF-α (H052-1-2), IL-6 (H007-1-2), IL-1β (H002-1-1), and IFN-r (H024-1-2). All reagents were purchased from the Nanjing Jiancheng Bioengineering Institute, and the testing operations were as described in the instructions.

### 2.6 16S RNA microbial sequencing

The fresh intestinal contents of the mice were collected, quickly frozen in liquid nitrogen and then stored in at −80°C. Bacterial DNA was extracted using a DNA rapid extraction kit, and a paired-end 16sRNA gene library was used for library construction and analysis. The V3+V4 region of the 16S rRNA gene was amplified using PCR and subsequently sequenced on the Illumina Hiseq 2500 sequencing platform using the PE250 mode (2 × 250 bp paired-end) (Biomarker Technologies Corp., China). In addition, the BMKCloud platform (www.BMKCloud) was used for the data analysis.

### 2.7 Colon histological staining

The colon was stained using hematoxylin-eosin staining (H&E). In short, fresh intestinal tissue was washed with PBS and then fixed with 4% paraformaldehyde. Frozen sections that were 10 µm thick were then constructed. Fresh sections were washed with ultrapure water, stained with hematoxylin for 30 seconds, and then stained with eosin for 30 seconds. After the samples were dried, they were photographed using a light microscope (Leica Co.). For the pathological evaluation, the HE-stained sections were scored as previously reported (14).

### 2.8 Statistical analysis

Data are expressed as the mean ± standard error of the mean (SEM). Furthermore, significant differences were determined by performing a t-test with least significant difference (LSD) *post hoc* tests, and statistical significance was set at p < 0.05.

## 3 Results

### 3.1 Mannose reduced the symptoms of DSS-induced colitis in mice

**Figure 1:**
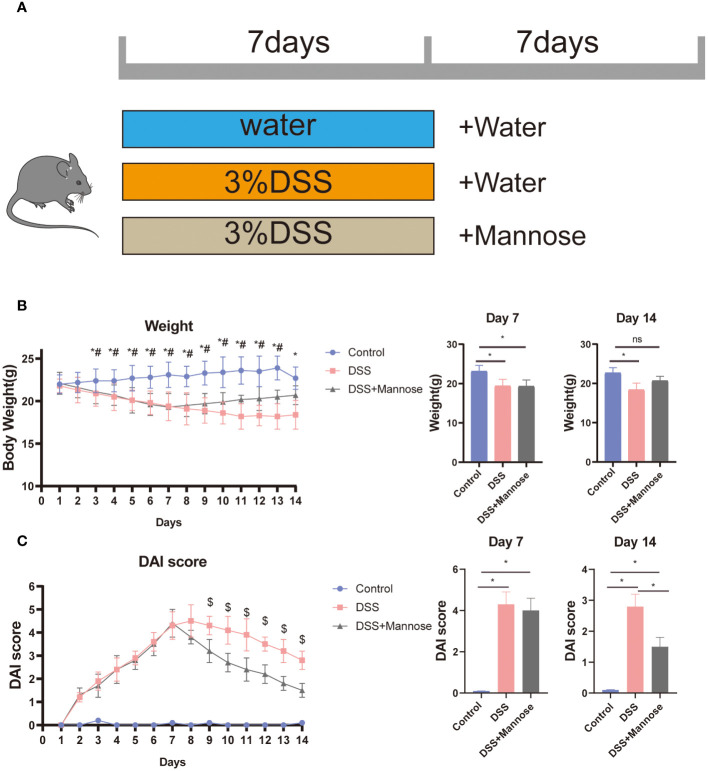
**(A)** Colitis mouse feeding model chart; **(B)** colitis mouse weight trend chart; and **(C)** colitis mouse DAI score chart. Significant differences are indicated as *p-value < 0.05. n=3-6/group. #P-value < 0.05, comparison between control and DSS+mannose.

To determine the therapeutic effect of mannose on DSS-induced colitis in mice, we used DSS to induce the mice for 7 days. We then established the DSS-induced colitis model. Mannose was further administered orally for 7 days, and its therapeutic effect on colitis was observed (**Figure 1A**). The results showed that compared with the control group, the mice in the DSS group showed an obvious weight loss trend after being continuously induced with DSS for 7 days. On the 7th day, it was statistically found that the weights of the mice with the DSS-induced colitis were significantly lower than that of the control group (P < 0.05) (**Figure 1B**), and the body weights on day 14 were also significantly lower than that of the control group. The results demonstrated that the DSS-induced colitis model was effective and significantly reduced the body weights of the mice. Additionally, 7 days after the colitis mouse model was induced using DSS, the body weights of the mice that had been orally administered mannose were significantly higher than that of the DSS group, indicating that mannose alleviated the DSS-induced weight loss in the mice. In addition, we calculated the disease activity index (DAI) scores of the mice during the experimental period. The results also showed that after 7 days in the DSS-induced mice, the DSS group significantly improved their DAI scores compared with the control group (P < 0.05). Subsequently, 7 days after the oral administration of mannose, the DAI scores of the mice in the mannose group were significantly lower than that of the mice in the DSS group (P < 0.05) (**Figure 1C**). The results indicated that the mannose had a significant therapeutic effect on DSS-induced colitis in mice.

**Figure 2:**
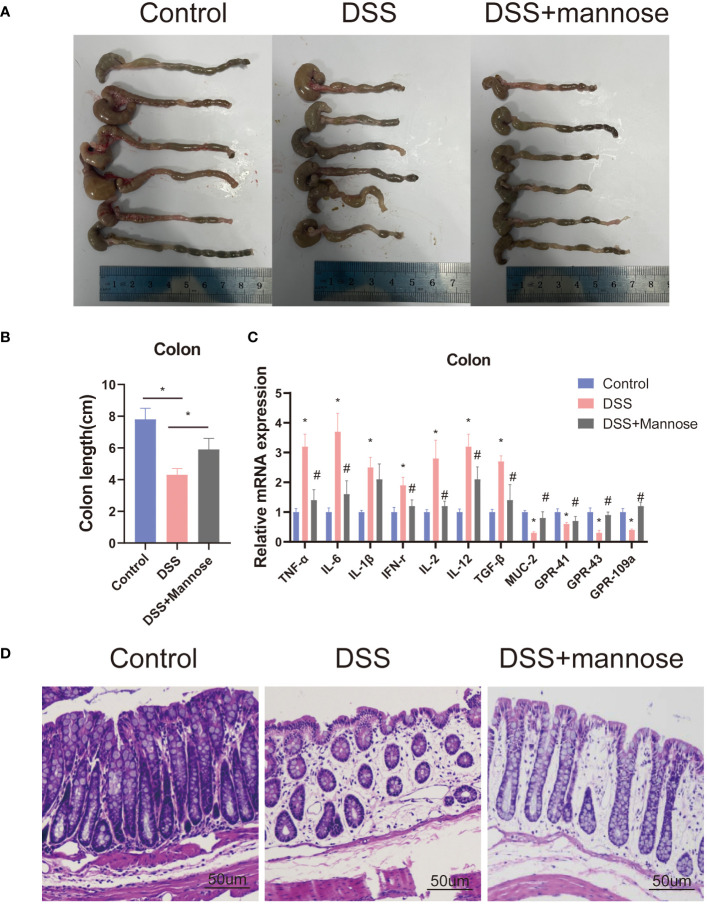
Phenotype of mice with colitis: **(A)** representative pictures of the colons of mice with colitis; **(B)** statistical diagram of the colon lengths of mice with colitis; **(C)** expression levels of the inflammatory genes in the colons of mice; and **(D)** representative pictures of the HE staining of the mouse colons. Significant differences are indicated as *p-value < 0.05. n=3-6/group. #P-value < 0.05, comparison between control and DSS+mannose.

To further observe the therapeutic effect of mannose on DSS-induced colitis in mice, we dissected the mice 14 days later and observed the colon lengths. The results showed that DSS significantly reduced the colon lengths of the mice (P < 0.05), while this result was significantly reversed after mannose treatment. The colons of the mice in the mannose group were significantly longer than those of the mice in the DSS group (P < 0.05) (**Figures 2A, B**). In addition, we stained the colon tissues of the three groups of mice and found that the colon walls of the control mice were clear, the villi were normal in shape, the epitheliums were intact, the glands were neatly arranged, the crypts were normal, the goblet cells were abundant, and there was no inflammatory cell infiltration. After colonic epithelial necrosis and shedding of the mice in the DSS group, the intestinal villi were abnormally atrophic, the intestinal walls became thinner, the glands were disordered, ulcer symptoms were severe, and a large number of inflammatory cells infiltrated the mucosal muscle layer. The colonic walls of the mice in the mannose group were clear and complete, and the villi shapes could be distinguished. There was local swelling of villi, a small amount of inflammatory cell infiltration, inconsistent gland arrangement, and rare ulcer symptoms (**Figure 2C**). These morphological results indicated that mannose had a therapeutic effect on DSS-induced colitis in mice, and its mechanism may have been the inhibition of intestinal inflammation and maintenance of the intestinal epithelial barrier function.

### 3.2 Mannose reduced the intestinal or serum inflammatory factor levels

**Figure 3:**
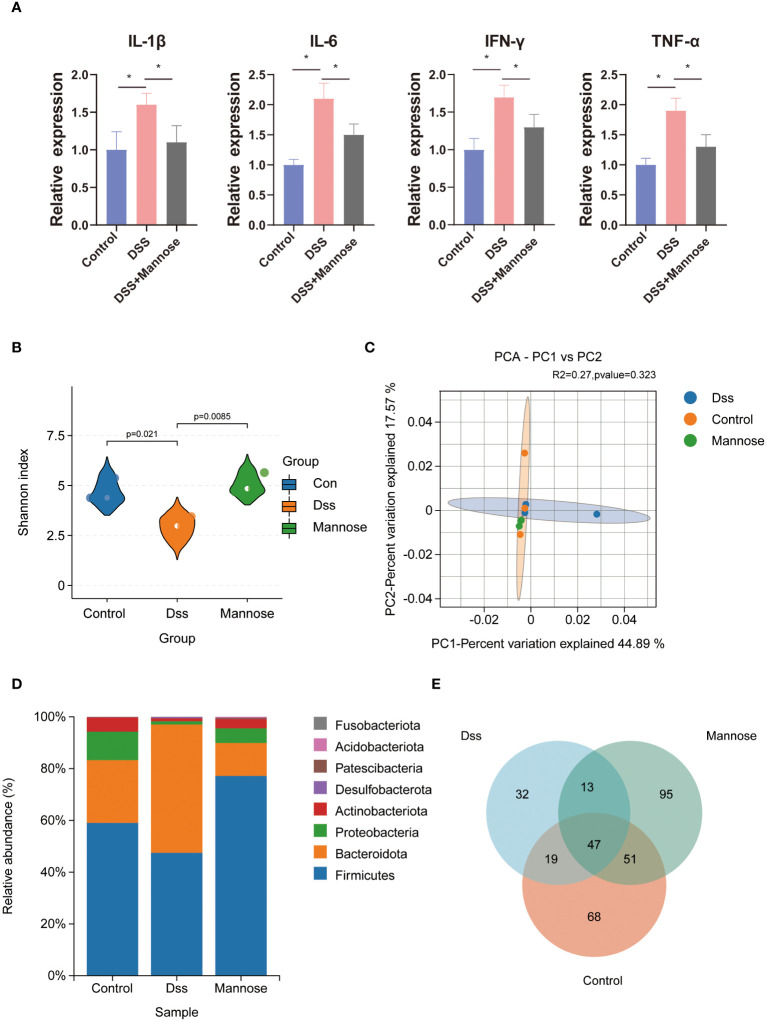
Mouse serology and microbial sequencing: **(A)** expression of inflammatory factors in the serum of mice; **(B)** alpha diversity of the intestinal microorganisms; **(C)** β diversity of the intestinal microorganisms; **(D)** differences in intestinal microbial phyla levels; and **(E)** intestinal microorganisms of the three groups of the mice Venn diagram. Significant differences are indicated as *p-value < 0.05.

To explore the mechanism of mannose on the colon inflammatory response of the mice, we extracted colon epithelial cells from the mice and further quantified the expression levels of related inflammatory genes. The results showed that the expression levels of colon inflammatory genes in the DSS group were significantly higher than those in the control group. TNF-α, IL-6, IL-1β, IFN-r, IL-2, IL-12, and TGF-β had all increased (P < 0.05), and the mannose treatment significantly alleviated this phenomenon. The mannose treatment significantly reduced the expression levels of inflammatory factors compared with the DSS group (**Figure 2D**). In addition, we further examined the expression level of the Muc2 gene in the colon epithelial cells and found that mannose reversed the inhibitory effect of DSS on the Muc2 gene in the mice. The results indicated that the therapeutic effect of mannose on colitis may have been due to a reduction in the expression of intestinal inflammatory genes that maintained the normal barrier structure of the intestine. Next, we further quantified the serum inflammatory factors in the mice and found that the expression levels of serum inflammatory factors (TNF-α, IL-6, IL-1β, and IFN-r) in the mice induced by DSS were significantly higher than those in the control group (P < 0.05). In contrast, the mannose treatment reduced the expression levels of related inflammatory factors (**Figure 3A**). This suggests that mannose may have antagonized the DSS-induced colon inflammatory response by reducing the intestinal or serum inflammatory factor levels.

### 3.3 Mannose may regulate the composition of intestinal microorganisms to exert anti-inflammatory effects

**Figure 4:**
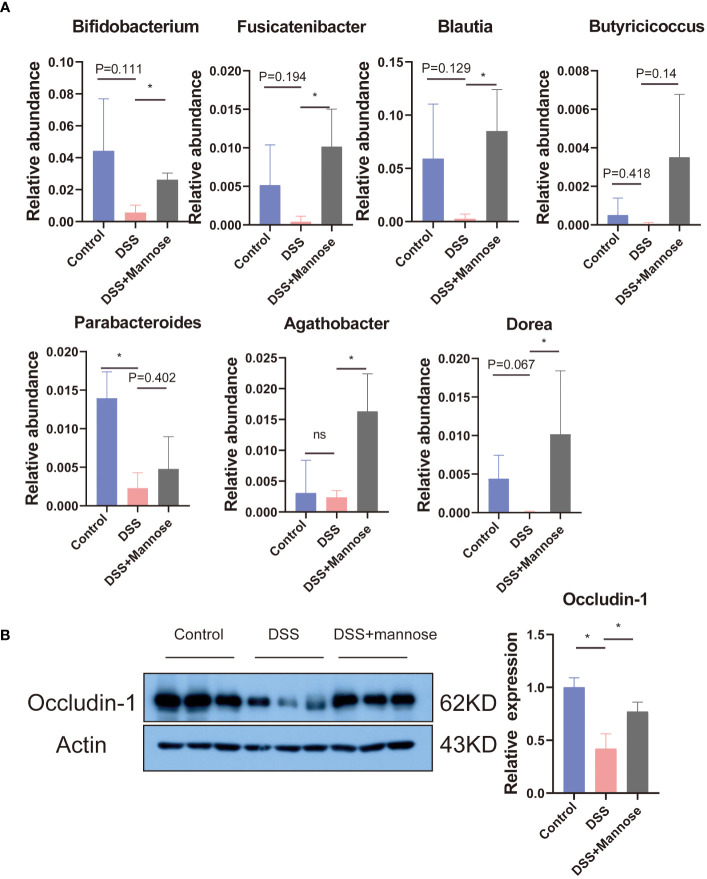
**(A)** Differences in the intestinal microbial bacterial abundances, and **(B)** the colon Occludin-1 protein expression. Significant differences are indicated as *p-value < 0.05. n=3-6/group.

To further explore the anti-colitis mechanism of mannose, we collected the colon contents of the mice and performed 16S RNA sequencing analyses. The results showed that after the DSS-induced colitis in mice, the composition of the intestinal microorganisms in the mice changed. The alpha diversity of the intestinal microorganisms was significantly lower than that in the control group (P < 0.05). Mannose treatment reversed this change, and the intestinal microbial alpha diversity of the mice that were orally administered mannose was significantly higher than that of the mice in the DSS group (P < 0.05) (**Figure 3B**). However, we did not find significant differences in the intestinal microbial beta diversities of the mice in the control, DSS, and mannose groups (**Figure 3C**). Subsequently, it was observed that there were significant differences in the phyla of the intestinal microorganisms of the three groups of mice. Compared with the control group, the Firmicutes phylum in the intestinal microorganisms of the mice in the DSS group decreased, while the Bacteroidetes phylum increased, and the Bacteroidetes phylum increased in the mannose group. The phylum level compositions of the intestinal microbiota of the mice were similar to that of the control group (**Figure 3D**). We further observed the differences in the genus level of the intestinal microorganisms among the three groups of mice and found that a total of 185 species of bacteria were detected in the control group, a total of 111 species of bacteria were detected in the DSS group, and a total of 206 species of bacteria were detected in the mannose group. A total of 47 bacterial genera were shared among the three groups of mice. The control group had 68 unique bacterial species, the DSS group had 32 unique bacterial species, and the mannose group had 95 unique bacterial species. The results indicated that mannose modulated the composition and abundance of intestinal microorganisms in the mice, and changes in the intestinal flora are a possible mechanism of how mannose treats colitis. To obtain a clearer understanding of the regulatory effect of mannose on mouse microorganisms, we closely examined the differences in the bacterial genera among the three groups of mice. The results showed that compared with the DSS group, the abundances of Bifidobacterium, Fusicatenibacter, Blautia, Agathobacter, Dorea, and other bacteria in the intestinal microbiota of the mannose-treated mice were significantly increased (P < 0.05) (**Figure 4A**). However, we did not observe differences in the ratio of Bacteroidetes to Firmicutes among the three groups(P < 0.05) (**Supplementary Figure S1A**). These findings suggest that mannose maintained the intestinal barrier function by regulating the abundance of intestinal probiotics.

We then further explored the changes in the intestinal barrier function. We compared the changes in the intestinal barrier function among the three groups by detecting the colon epithelial tight junction protein (Occludin-1). The results showed that compared with the control group, the expression levels of the Occludin-1 protein in the colon of the mice induced with DSS were significantly reduced (P < 0.05). Mannose treatment completely reversed this phenomenon. Compared with the DSS-induced mice, the expression of the Occludin-1 protein was significantly increased (P < 0.05) (**Figure 4B**).

### 3.4 Fecal transplantations in the mannose-treated mice improved colitis

**Figure 5:**
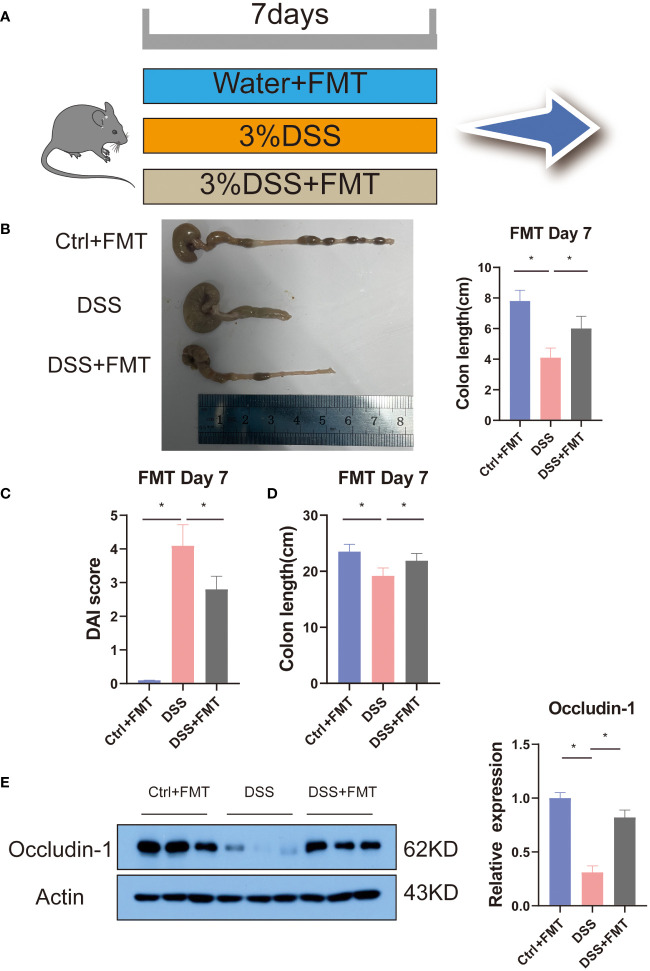
Fecal bacteria transplant model diagram: **(A)** schematic of the fecal bacteria transplantation; **(B)** representative pictures of the mouse colons; **(C)** and **(D)** are diagrams of the mouse colon lengths and colon DAI scores, respectively; and **(E)** expression of the Occludin-1 protein in the colons of mice transplanted with fecal bacteria. Significant differences are indicated as *p-value < 0.05. n=3-6/group.

**Figure 6:**
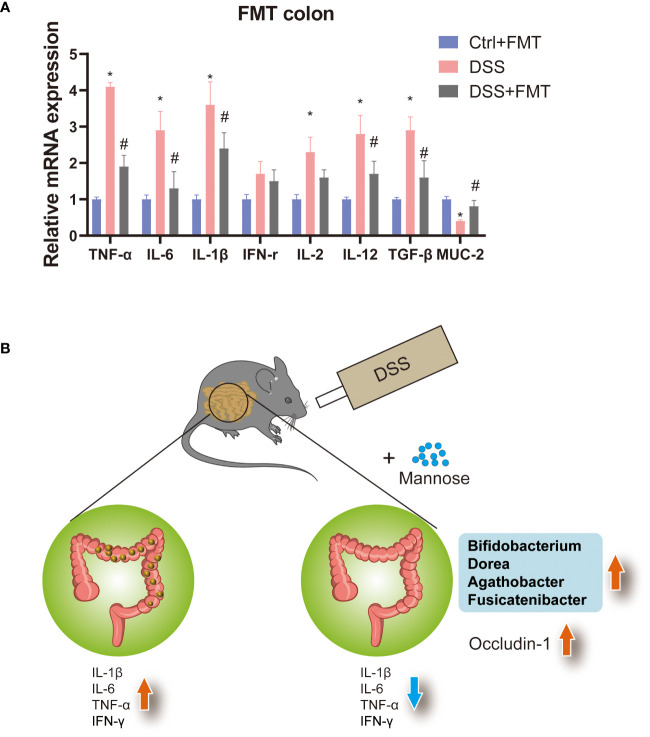
**(A)** Expression levels of inflammatory genes in the colons of mice transplanted with fecal bacteria, and **(B)** model diagram of the mannose treatment of colitis. Significant differences are indicated as *p-value < 0.05. n=3-6/group. #P-value < 0.05, comparison between control and DSS+mannose.

Previous studies have shown that mannose alleviates colitis in mice by regulating the intestinal microbiota of mice. To verify this idea, we used the feces of mice that were orally administered mannose as material for fecal microbiota transplantation (FMT). We then performed FMT on the mice (**Figure 5A**). The results showed that at the end of the 7-day experiment, the lengths of the colons of the mice were significantly shorter in the DSS group than in the control group(P < 0.05), while the lengths of the colons of the DSS+FMT group were significantly longer than those of the mice with DSS-induced colitis (P < 0.05) (**Figure 5B**). This suggests that the fecal bacteria transplanted from the mannose-treated mice could be used to treat DSS in mice. We further measured the mouse body weights and disease activity scores (DAI score), and the results were consistent. The weights of the mice with DSS-induced colitis were significantly reduced, and the DAI scores were significantly increased (P < 0.05). However, the fecal bacteria transplantation in mice increased their body weights, and their DAI scores decreased (P < 0.05) (**Figures 5C, D**). This further demonstrates that mannose alleviated colitis in mice by regulating the intestinal microbiota. In addition, to verify the improvement effect of fecal bacterial transplantation on the intestinal barrier function, we quantified the Occludin-1 protein in the colons of the mice with fecal bacterial transplantation. The results showed that the expression levels of Occludin-1 were significantly higher in the mice in the DSS group than those in the control group. This malignant change was reversed in mice transplanted with fecal bacteria (P < 0.05) (**Figure 5E**). This indicates that the intestinal microorganisms after FMT improved the damaging effect of DSS on the colon barrier function of the mice. To this end, we tested our conjecture again by detecting changes in the expression levels of colon inflammatory genes. The results showed that the expression levels of colon inflammatory genes in the DSS group were significantly higher than those in the control group (TNF-α, IL-6, IL-1β, IFN-r, IL-2, IL-12, TGF-β) (P < 0.05), while the fecal bacteria transplanted mice had significantly inhibited expression of inflammatory genes (**Figure 6A**). Consistently, we found that inflammatory factors (IL-1β and TNF-α) in serum were significantly lower in the fecal transplant group than in the DSS group (P < 0.05) (**Supplementary Figures S1B, C**). The results indicated that mannose improved mouse colitis by regulating the intestinal microbiota of the mice (**Figure 6B**).

## 4 Discussion

Mannose is a six-carbon monosaccharide that is metabolized by the body through absorption in the small intestine (15). In this study, we found that the oral administration of mannose alleviated DSS-induced colitis in mice, which involved reducing the expression levels of colonic inflammatory genes and serum inflammatory factors. In addition, we found that mannose rescued the inhibitory effect of DSS on the colonic tight junction protein Occludin-1. Tight junction proteins play a crucial role in the formation and maintenance of the intestinal epithelial barrier. The occurrence of IBD involves a disruption in the tight junctions and imbalances of the epithelial cell permeability (16, 17). Our study showed that mannose prevented a decrease in the tight junction protein expression induced by DSS administration, thereby alleviating colitis symptoms. This result was consistent with previous research results (2, 18–20). Relevant scholars have suggested that the therapeutic effect of mannose on DSS-induced colitis may be related to the regulation of macrophage polarization in the colon that may involve the release of TNF-α and pathological endoplasmic reticulum stress of intestinal epithelial cells (20).

In addition, we sequenced the cecal contents and found that mannose modulated the composition of intestinal microorganisms, including the bacterial abundance and alpha diversity. There were increased abundances of Bifidobacterium, Fusicatenibacter, Blautia, Agathobacter, and Dorea. These probiotics have been reported to improve intestinal inflammation (21, 22). Therefore, we hypothesized that the therapeutic effect of mannose on colitis may be related to its modulation of intestinal microorganisms. To test our hypothesis, we performed fecal bacterial transplantation using the feces of mannose-treated mice. In mice with DSS-induced colitis, fecal bacteria transplanted from the mannose-treated mice had an anti-colitis effect. Specifically, fecal bacteria transplanted into mice reduced intestinal inflammatory gene expression and increased the Occludin-1 protein level. These results indicate that mannose enhanced the intestinal immune barrier function through intestinal microbiota and alleviated DSS-induced colitis in mice. Previous researchers have found that mannose can exert anti-obesity effects by regulating intestinal microorganisms (10, 23). In this study, we found that mannose treatment of DSS-induced colitis in mice may have been related to its modulation of intestinal microorganisms. In short, mannose modulation of intestinal microbiota is a feasible strategy for treating colitis.

The primary feature of IBD is a disruption of intestinal homeostasis, including dysregulated immune responses, eating disorders, genetic susceptibility, intestinal microbiota imbalance, and barrier damage (4, 5). Gut microbes have been shown to modulate intestinal immunity, eating disorders, and genetic susceptibility and improve intestinal barrier function (24–27 al., 2023). Therefore, we have reason to believe that mannose’s regulation of intestinal microorganisms may be related to the alleviation of colitis. DSS is a chemical substance that stimulates the intestinal epithelium and causes the destruction of the mucosal-epithelial barrier, resulting in an increase in intestinal epithelial permeability. This causes macromolecular substances (including toxic molecules and metabolic wastes) to pass through the intestine and enter the blood, causing local or systemic symptoms (28). Intestinal microorganisms serve as a protective film adherent to the intestinal mucosa or intestinal epithelial surface that can reduce the infiltration of toxic chemical substances into the intestinal epithelium and reduce the release of inflammatory mediators within the intestinal epithelium (20). This is consistent with the view that related drugs can alleviate colitis (29–31). This explains the feasibility that the oral administration of gut microbiota (FMT) with mannitol can also alleviate DSS-induced colitis in mice. Another critical factor for intestinal barrier function is the expression level of the intestinal epithelial tight junction proteins. The most important is the Occludin-1 protein that plays a key role in the intestinal barrier (32). We found that fecal bacterial transplantation can increase the expression of the colonic Occludin-1 protein in mice with DSS-induced colitis. This may be due to the interaction between the products of the intestinal metabolism of probiotics after transplantation and the intestinal epithelium. This also explains the beneficial effects of fecal transplantation.

## 5 Conclusion

In this study, we confirmed that mannose can alleviate DSS-induced colitis in mice, and its mechanism is related to inhibiting the expression of intestinal inflammatory genes and maintaining the intestinal barrier function. In addition, we found that this therapeutic effect may have been due to mannose regulating the intestinal microbiota. We further confirmed that mannose enhanced the intestinal immune barrier function by regulating intestinal microbiota through fecal bacterial transplantation in mice that were orally administered mannose. This then alleviated DSS-induced colitis in mice.

## Data availability statement

The data presented in the study are deposited in the NCBI SRA repository, accession number PRJNA1083203.

## Ethics statement

The animal studies were approved by the Experimental Animal Ethical Committee of Jinan University. The studies were conducted in accordance with the local legislation and institutional requirements. Written informed consent was obtained from the owners for the participation of their animals in this study.

## Author contributions

YY: Data curation, Formal analysis, Investigation, Resources, Writing – original draft, Writing – review & editing. QM: Data curation, Formal analysis, Investigation, Resources, Software, Writing – review & editing. QW: Data curation, Investigation, Software, Writing – review & editing. LZ: Validation, Writing – original draft, Writing – review & editing. HL: Formal analysis, Resources, Software, Writing – original draft, Methodology. YC: Conceptualization, Data curation, Investigation, Project administration, Resources, Supervision, Validation, Writing – original draft, Writing – review & editing, Funding acquisition.

## Supplementary material

Supplementary Figure 1
**(A)** The ratio of Bacteroidetes and Firmicutes; **(B, C)** expression of inflammatory factors in the serum of mice transplanted with fecal bacteria.
