# Supplementary figures and images for: Mannose enhances intestinal immune barrier function and dextran sulfate sodium salt-induced colitis in mice by regulating intestinal microbiota
Source: Front Immunol. 2024 Mar 11;15:1365457. doi: 10.3389/fimmu.2024.1365457 (PMC10961387; doi:10.3389/fimmu.2024.1365457)

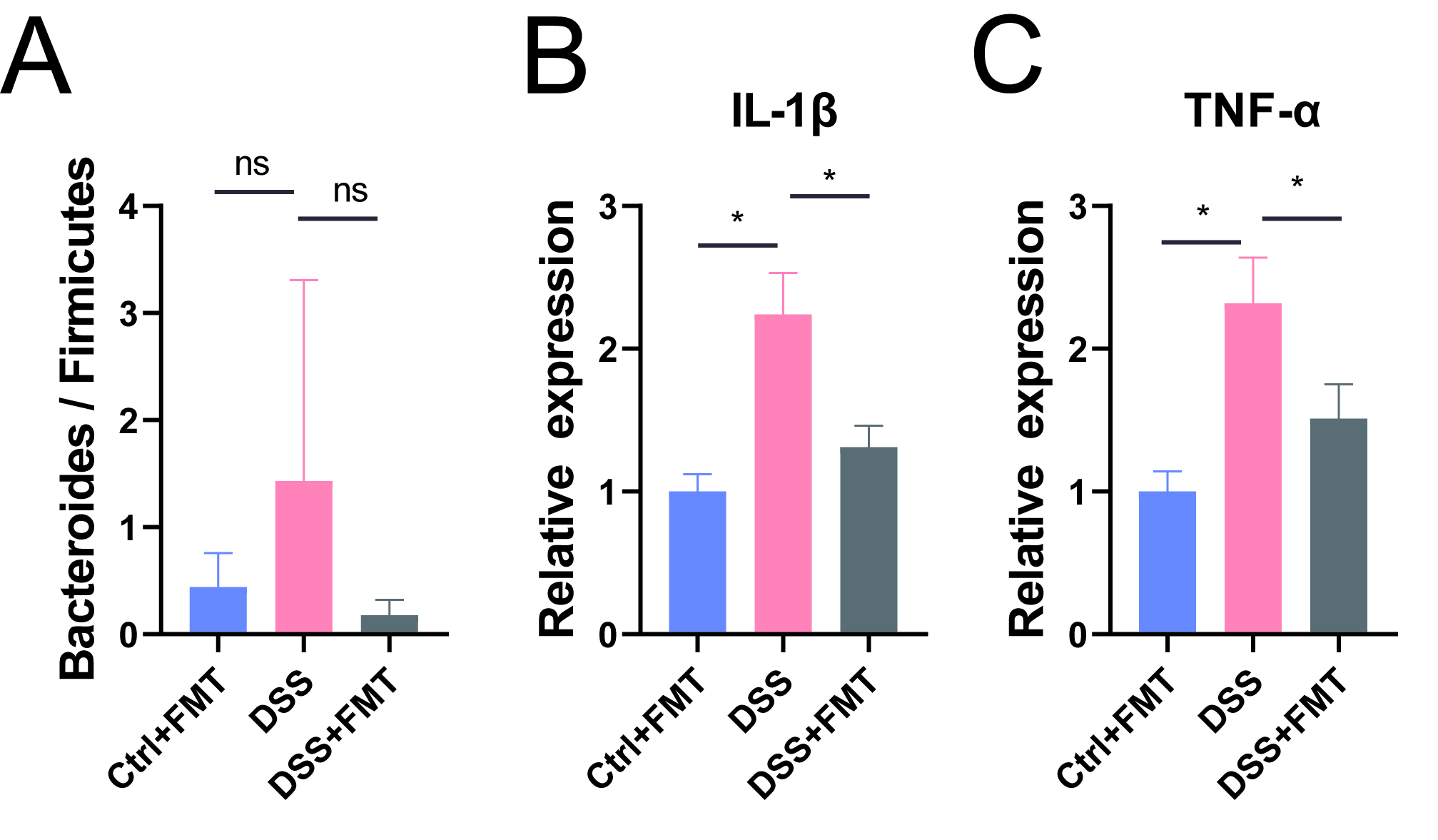

Supplement: Supplementary Figure 1 — (A) The ratio of Bacteroidetes and Firmicutes; (B, C) expression of inflammatory factors in the serum of mice transplanted with fecal bacteria. [file Image_1.tif]
